# A Method of Capping Exposed Pulps, with Cases in Practice

**Published:** 1880-11

**Authors:** M. S. Jobson

**Affiliations:** Perry, Georgia


### ARTICLE III.

### *A Long Practiced and Successful Method of Capping Exposed Pulps, with Cases in Practice.*

BY M. S. JOBSON, PERRY, GEORGIA.

[Read before the Georgia State Dental Society.]

Since cements have come into use in filling teeth the old way of capping exposed pulps with gold and other material, which was practiced to a very limited extent, has come to be a thing of the past, and by the use of cements the operation has been brought to a basis of safety and certainty. Capping with gold required the nicest manipulation, was very tedious and very uncertain in results, and was consequently attempted by but very few; but with the cements the work is short and simple, and with it nearly every exposed pulp can be saved.

Extirpation should now cease. We should save the teeth of the people with the nerves in them, and there is no reason why every good operator should not succeed in doing so, and he who does succeed will have abundant cause for self-gratulation, and will save his patients much pain, time and money.

Other methods, differing some from the following, have appeared in print and been noted, but I do not think that by them success is so sure as by this. For the best results, the tooth should be dealt with very tenderly until the nerve is safely under the capping. Excavate in a way that will give no pain, where that is possible, and in syringing use water of a temperature that will give no shock. In the majority of cases every particle of decay should be removed from over and around the pulp, however large that may make the exposure. The floor of the cavity immediately around the exposure make flat and smooth in every case where it is possible. The membrane of the pulp is then clipped, and as much blood let out as will come, and where the pulp has been much irritated, or is very red and the blood comes very slow, it should be encouraged by tepid water. The rubber dam is now applied, and the cavity dried and flowed with carbolic acid. In every case that will do for immediate capping; the tooth will be perfectly easy and comfortable after the hemorrhage and application of acid, and where it is not a slight quantity of carbolic acid on a small pellet of cotton should be lain in light contact with the nerve and a loose piece of dry cotton placed over that and kept there some minutes or hours, as may be necessary to bring the tooth to a perfectly comfortable condition, and until then no capping or stopping should be put in. Where the tooth is not quickly relieved of all pain the capping should be deferred several hours, or until the next day.

Over the exposed pulp and on the smooth floor around it lay one to four thicknesses of strong, bibulous paper, cut to proper size and shape, so it may not wrinkle when the cement is carried down against it. Japanese or French bibulous paper will not do for this; the cement may pass through the first, and the latter is liable to break under it. If all the carbolic acid has now been absorbed the cavity is again flowed with it. The cement is then mixed thin and a small batch carried into the cavity with spatula, and down to place on the paper and floor of the cavity with a piece of spunk or soft bibulous poper, particular care being had not to carry the cement down enough to bear or press in the least upon the pulp, but enough that there may be no space between the pulp and capping. If the capping bears upon the pulp, failure will certainly result. This first batch of cement being thin where it is carried down to place, unites, to some extent, with the carbolic acid, and the paper or spunk with which it is carried to place absorbs the surplus carbolic acid and liquid of the cement. If the first pellet does not dry it sufficiently, as many as may be necessary to do so should be used, thus hastening the setting, and the cement thereby becoming harder. More cement is then carried in and to place in the same manner, until the capping is of thickness desired or the cavity full, and the work is done, in so far as capping. In most cases the capping gives little or no pain, and if the pulp and surroundings of the tooth are in proper condition to be treated this way success may be expected in forty-nine cases of fifty.

In almost every favorable case the pulp, under this treatment, will recede and throw out bony tissue or be tiself converted into tooth structure. I have never used any of the advertised non-irritant cements, or those prepared especially for this purpose. Have used Houghton's Os-artificial, Smith's Oxychloride of Zinc, large and small packages, Cement Plombe, Guillois' Cement, and Agate Cement, all with equal success. I have been treating exposed pulps in this way since a few months after I began the practice of dentistry, and now after thirteen years' experience, I am prepared to say that there is no way of treating such teeth that approaches this in safe, certain and satisfactory results.

### CASES IN PRACTICE.

*Case* 1.—In 1872, while filling a tooth for Miss P., I was particularly asked not to touch the second left inferior molar, as it was exceedingly sensitive, and would have to be taken out. The tooth was largely decayed on grinding surface, but elsewhere and otherwise was in perfect condition. The pulp lay bare in the cavity. After persuasion she consented to let me see if I could fix it without too much pain. In less than two hours I had the tooth completed with a gold filling. A little girl stood on an ottoman by my chair and did the malleting with a steel mallet. Capping nor filling gave any pain, and she declared some months after it was the best tooth she had.

*Case* 2.—In 1872, Mr. W. complained of the second left inferior molar troubling him. I found a portion of the pulp dead. Removed dead part, capped the remainder and filled with amalgam. This tooth has been and is to day in a perfect condition.

*Case* 3.—In 1874, Mr. C. called, with severe toothache. Pulp exposed and very red. Removed all decay; clipped pulp membrane, and by aid of tepid water, drew all the blood that would come. Applied rubber dam, flowed cavity with carbolic acid, placed bibulous paper over pulp, and filled cavity up with cement. Six or eight months after took out what cement remained and no pulp was visible. Cut up into the tooth as far as the neck without giving any pain or finding the nerve, or any indication of one. Filled with gold. If I remember correctly, the tooth had not given a tinge of pain from the moment I dismissed patient after capping. The operation throughout was perfectly satisfactory, and so far as I am aware the tooth remains so to this day.

*Case* 4.—In 1874, Mr. M. (now Dr. M.) complained of a tooth that had been filled with amalgam. After using, in vain, every means of relief I could, I removed the amalgam, which I found was in contact with the nerve. Blood flowed immediately and for several minutes, and I encouraged with tepid water. After hemorrhage ceased I placed a small pellet of cotton saturated with carbolic acid in contact with the pulp, and a loose piece of dry cotton over that and dismissed him. The tooth in a few hours got into a comfortable condition, and the next day or day after I capped and filled up cavity with capping. The tooth remained in a perfectly satisfactory condition, and after a few months excavated a portion of the cement and filled with gold. The cavity embraced the anterior proximal and grinding surfaces. The tooth was in the best possible condition two weeks ago.

*Case* 5.—In 1875, Miss C. came with the palatine wall of the second right superior bicuspid broken away. The tooth had large gold fillings in both proximal surfaces, but that only in the posterior came out. In proceeding to excavate for refilling I found the pulp had been capped. The capping came out in one mass, and I found the pulp chamber vacant. I drew out with a broach a part of the pulp alive and occupying the end of the fang. The tooth, under and around the capping was of a brownish hue, as was also the cement, but both were sound and hard. There was not the slightest disagreeable odor in the pulp chamber. The tooth had never given any trouble since it had been filled. I made no note of this capping, but found by my registering ledger had filled the tooth in September, 1869.

*Case* 6.—August, 1875, Miss E., first right inferior molar; large decay, embracing buccal and crown surfaces. The pulp of this tooth had enlarged or grown up to near the orifice of the cavity. Excised down to surface of its chamber with comparatively little pain. After bleeding, which was profuse, excavated and filled. The tooth had not troubled her much. In 1877, she complained some of the tooth, and I removed the filling, somewhat against her will. I do not remember whether the pulp bled any of itself after removal of capping, but I drew from it all that would come, capped and filled again, and there has been no trouble since. I found on removing the capping in this tooth the pulp had contracted, or receede, and the chamber between it and capping was vacant.

*Case* 7.—March 27th, 1879, Mrs. P., right superior lateral incisor—corner broken away. In excavating for anchorage exposed the pulp; capped and filled cavity with capping in usual way. June 27th, 1879, removed all the cement, pulp had disappeared, the floor and walls of the cavity clear and white, and not sensitive; built tooth out with gold.

*Case* 8.—April 7th, 1880, Mr. S., first left superior molar; excavated, bled exposed pulp and capped in usual way. Three days after he was in town, and was to call at 12 o'clock, to have "something done" with his tooth, as it was "still troubling" him. Saw nothing more of him until 6th instant. The tooth, he said, got perfectly easy, and had been so satisfactory since he is anxious to get back to have other sensitive ones fixed.

---

